# Internet-based surveillance of Influenza-like-illness in the UK during the 2009 H1N1 influenza pandemic

**DOI:** 10.1186/1471-2458-10-650

**Published:** 2010-10-27

**Authors:** Natasha L Tilston, Ken TD Eames, Daniela Paolotti, Toby Ealden, W John Edmunds

**Affiliations:** 1 London School of Hygiene and Tropical Medicine, Keppel Street, London, WC1E 7HT, UK; 2 ISI Foundation, Viale Settimio Severo, 65 10133 Torino, Italy

## Abstract

**Background:**

Internet-based surveillance systems to monitor influenza-like illness (ILI) have advantages over traditional (physician-based) reporting systems, as they can potentially monitor a wider range of cases (i.e. including those that do not seek care). However, the requirement for participants to have internet access and to actively participate calls into question the representativeness of the data. Such systems have been in place in a number of European countries over the last few years, and in July 2009 this was extended to the UK. Here we present results of this survey with the aim of assessing the reliability of the data, and to evaluate methods to correct for possible biases.

**Methods:**

Internet-based monitoring of ILI was launched near the peak of the first wave of the UK H1N1v influenza pandemic. We compared the recorded ILI incidence with physician-recorded incidence and an estimate of the true number of cases over the course of the epidemic. We also compared overall attack rates. The effect of using different ILI definitions and alternative denominator assumptions on incidence estimates was explored.

**Results:**

The crude incidence measured by the internet-based system appears to be influenced by individuals who participated only once in the survey and who appeared more likely to be ill. This distorted the overall incidence trend. Concentrating on individuals who reported more than once results in a time series of ILI incidence that matches the trend of case estimates reasonably closely, with a correlation of 0.713 (P-value: 0.0001, 95% CI: 0.435, 0.867). Indeed, the internet-based system appears to give a better estimate of the relative height of the two waves of the UK pandemic than the physician-recorded incidence. The overall attack rate is, however, higher than other estimates, at about 16% when compared with a model-based estimate of 6%.

**Conclusion:**

Internet-based monitoring of ILI can capture the trends in case numbers if appropriate weighting is used to correct for differential response. The overall level of incidence is, however, difficult to measure. Internet-based systems may be a useful adjunct to existing ILI surveillance systems as they capture cases that do not necessarily contact health care. However, further research is required before they can be used to accurately assess the absolute level of incidence in the community.

## Background

Every year influenza A viruses cause epidemics leading to high morbidity and mortality - approximately 3 to 5 million cases and between 250,000 and 500,000 deaths worldwide [1]. The majority of complications usually occur in the young, elderly and the immunologically compromised. Although immunity from the virus may be long term, this immunity is subtype and strain specific which means that as long as the virus retains its ability to mutate rapidly recurring outbreaks will continue to be seen [1-3].

On the 12^th ^of June 2009, the WHO alerted the world of an influenza pandemic caused by a novel H1N1 influenza strain. The UK epidemic was characterised by two peaks: one in late July, and another in late October [4]. Traditional influenza monitoring methods have relied on sentinel networks of physicians to diagnose and report ILI (influenza-like-illness) [5]. These systems have been the mainstay of influenza surveillance for many years. However, they have a number of potential drawbacks, particularly during a pandemic, as they require individuals to attend physicians when they are ill. An unknown proportion of cases do not attend heath care, and this proportion may vary with age, sex or other social groups, and may change over the course of an epidemic, particularly if there are changes in levels of public concern, capacity problems, or mechanisms to divert patients away from physician's offices (such as was implemented in the UK during 2009). In response, internet-based surveillance has been suggested as a means of rapidly assessing the level of illness in the community. Two approaches have been implemented. First, people's internet browsing behaviours have been used as proxies for ILI incidence [6-13]. As these only record proxy measures of influenza, they are limited in their usefulness (they cannot, for instance, track health-care usage, or the clinical spectrum of cases). An alternative is to recruit members of the public to record, using an internet-based questionnaire, specific symptoms over time. That is, to recruit an internet-based cohort and survey patterns of illness and health care attendance in these individuals. Such systems have been implemented in Belgium and the Netherlands (under the name "Der Grote Griepmeting" - the Great Influenza Survey, GIS) in 2003 [14], Portugal ("Gripenet") in 2005 [15], and in Italy ("Influweb") in 2007 [16]. Results, in non-pandemic years, appear to be highly correlated temporally with those obtained through traditional surveillance methods [17-19], and the systems may be able to detect increased influenza activity more rapidly than GP-based surveillance [17-19]. The incidence recorded in these systems appears to be consistent between countries, and is consistently higher than that reported by GP-based systems [19]. However, it is not clear exactly what is being measured by the internet-based surveillance systems, as the sample is unlikely to be representative and it is not clear how to calculate the appropriate denominator (see below). We adapted these internet-based influenza monitoring systems to the UK in July 2009 (during the first wave of the UK pandemic of H1N1v [20]). Here we present data collected using this system. We examine different methods of calculating incidence from such a system, including examining different ways of defining influenza-like-illness. We compare our incidence estimates to the case estimates generated by the Health Protection Agency (HPA) [21], noting that these case estimates should themselves be treated with some caution, and with the ILI attack rates for England and Wales. The aim is to assess how well internet-based surveillance of Influenza performs when compared to a range of other measures.

## Methods

### Implementing the UK flusurvey

The UK flusurvey was launched in mid-July 2009. With the help of a publicity campaign involving television, radio, and newspaper coverage and word of mouth, over 5,000 participants were recruited in the first week. Participants were recruited from all parts of the UK (Additional file 1).

The study was approved by the London School of Hygiene and Tropical Medicine Ethics Committee (Application number 5530).

Registration for the UK flusurvey took place through the web page http://www.flusurvey.org.uk. Upon registration, following provision with a password-protected account, participants were requested to complete a background questionnaire. This covered age, gender, household size and composition, occupation, location of home and workplace, receipt of influenza vaccine during the 2008/2009 influenza season, and membership of a risk group (self-report of any of the following: diabetes, asthma, other chronic lung disease, immunocompromised, chronic heart disease, other chronic disease, pregnant - individuals reporting any of these are referred to hereafter as being in a "risk group"). As well as registering on their own behalf, participants were able to create accounts on behalf of other members of their family/household, thus enabling, for instance, parents to record data about their children.

Each week, whether or not they had symptoms, participants were asked to complete a symptoms questionnaire (see below) and a questionnaire about their social contacts. A questionnaire about vaccine uptake was added in autumn 2009, once the pandemic-specific vaccine became available. Each questionnaire was intended to take no more than a couple of minutes to complete.

An email newsletter was sent to participants each week to remind them to complete the symptoms questionnaire. To maintain participants' interest in the survey the weekly newsletters contained a summary of the latest influenza facts and news, and the flusurvey website was updated on a daily basis with items including the estimated incidence and the spatial distribution of cases being continuously updated.

### Symptoms questionnaire

The symptoms questionnaire asked participants to record which, if any, symptoms they had experienced recently. Participants were asked to select symptoms from a list, shown in Table 1.

**Table 1:** The list of symptoms used in the symptoms questionnaire.

1.	Blocked/runny nose
2.	Cough
3.	Sore throat
4.	Headache
5.	Muscle pain and/or joint pain
6.	Chest pain
7.	Stomach ache
8.	Diarrhoea
9.	Nausea
10.	Chills
11.	Weakness
12.	Eye irritation
13.	Fever
14.	No symptoms

Participants were asked to select any symptoms that applied to them.

Participants who reported the presence of symptoms were asked when their symptoms began and whether fever onset (if any) was rapid; they were asked about healthcare seeking behaviour - whether they consulted a medical professional and, if so, whether in person or by phone/internet, who they consulted, and when the consultation took place; they were asked whether they took any medication as a result of their symptoms, and, if so, when; they were asked whether they took time off work as a result of their symptoms and, if so, for how long they were off work. Participants were also asked whether they had encountered anyone with flu like symptoms in the past week.

### Sample used in the analysis

Here we analyse data collected via the symptoms questionnaire from the period 16/07/09 until 31/12/09. In all, 5,738 participants contributed 20,901 records to this dataset.

In an attempt to reduce the effect of individuals who only register and take part as a one-off response to their current symptoms we followed the method of Friesema *et al. *[17], by restricting our dataset to participants' second and subsequent reports (15,163 records). We compared estimates of incidence from this dataset with those produced using a third dataset that contained all reports made by participants who participated more than once (17,532 reports), and found that they were very similar. Here, therefore, we use the latter dataset, and will from now refer to this sample as the "censored sample".

### Demographic description of Flusurvey participants

**Figure 1:**
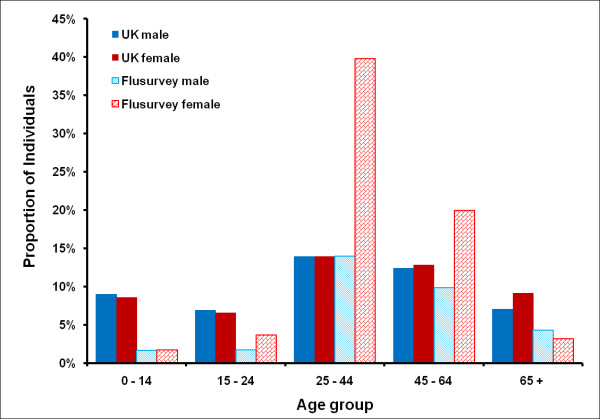
**Comparison of the flusurvey population in different age and gender categories with the UK population **[22].

The censored sample contains 2,369 participants who contributed 17,532 completed symptoms questionnaires. A comparison of this sample with the UK population [22] makes it clear that our sample is not demographically or geographically representative (Figure 1 and Table 2). 2% (49 out of 2369) did not report their age or sex. 67% of Flusurvey participants were women. People aged under 15 and over 60 are under-sampled; 88% were from England, London in particular being over-sampled.

**Table 2:** Comparison of the spatial distribution of our sample with the UK general population [22]

Region	Flusurvey Population (2369)	UK Population (61383200)
East Midlands	5%	7%
East of England	12%	9%
London	23%	12%
North East	2%	4%
North West	8%	11%
South Central	9%	7%
SE Coast	7%	7%
South West	8%	8%
West Midlands	5%	9%
Yorkshire & the Humber	8%	8%
Wales, Scotland & NI	11%	16%
Unknown	2%	-

**Figure 2:**
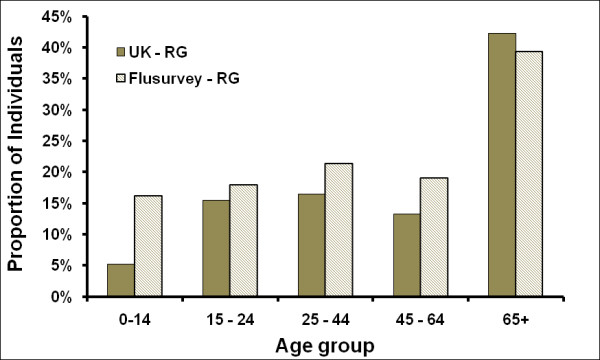
**Proportion of individuals in an influenza risk group, comparing the population of England with English Flusurvey participants **[22,23].

Flusurvey participants were similar, in terms of risk status, to the general population [23], with the exception of children (Figure 2). These differences could be partly explained by differences in definitions - particularly for asthma (respondents could well have reported mild and/or intermittent asthma which would not necessarily put them in a risk group, according to the definition usually used in the UK [24]). The Flusurvey seasonal flu vaccine uptake for last year in the under 65 risk group was about 40% when compared with the UK (47.1%) and in the 65+ age group it was about 68% compared to the UK (74%) [25].

### Weighting

Because of the demographic biases contained within our sample, in order to allow comparison between influenza statistics derived using the Flusurvey data with those estimated for the UK [21,23], we consider reweighting our sample in the analysis that follows. To do this we weight our sample according to age and gender. We split our sample into age categories < 1, 1 - 4, 5 - 14, 15 - 24, 25 - 44, 45 - 64 and 65+ to match the UK census age distribution. Each individual is assigned a weight according to the following formula [26]:

$${\text{W}}_{\text{i}}\;={\text{P}}_{\text{i}}{}^{\text{U}\text{K}}/{\text{P}}_{\text{i}}{}^{\text{F}\text{l}\text{u}\text{s}\text{u}\text{r}\text{v}\text{e}\text{y}}$$

Where,

W_i _= Weight of individual i

P_i_^UK ^= Proportion of the UK population in the same age and gender group as individual i

P_i_^Flusurvey ^= Proportion of the Flusurvey population in the same age and gender group as individual i

Analysis and calculations were carried out in Stata 11 and Excel 2007.

### Defining influenza-like-illness

In an ideal world it would be possible to use a simple list of symptoms to determine, with perfect sensitivity and specificity, whether someone had experienced an influenza infection. However, the symptoms of influenza are subject to too much variation from person to person, and there are too many other infections with similar symptoms. Therefore, instead, we seek to use the symptoms questionnaire to decide which participants had experienced ILI. This distinction between influenza itself and influenza-like-illness is one made by most public health bodies [27,28], in the absence of laboratory confirmation.

ILI can be determined using a symptoms list but, to further complicate matters, several different definitions were in use during the H1N1v pandemic. For example, the Health Protection Agency (HPA) defined ILI as "*Fever plus two or more of the following symptoms - cough, sore throat, runny nose, limb/joint pain, headache, vomiting/diarrhoea*" [27], whereas the US Centers for Disease Control and Prevention (CDC) defined it as "*Fever plus cough plus headache plus any of the following symptoms - runny nose, blocked nose, joint pain, muscle pain, weakness, sore throat, chest pain, abdominal pain, nasal congestion) *[28]. The GIS, on which the UK flusurvey was based, uses the following definition of ILI [17]: "a *sudden onset of fever with muscle pain accompanied by either a cough, sore throat or chest pain*".

We compare these three definitions to explore the effect they would have on estimated incidence of ILI. However, for comparability with previous work [19], and to allow data to be quickly compared from country to country, we will make use of the GIS definition of ILI unless stated otherwise.

Because not every participant recorded the date of symptom onset, and because of the difficulty in determining an appropriate denominator (see below), we measure ILI incidence in week j as the number of participants recording, in week j, symptoms consistent with ILI.

### Defining a denominator for incidence estimation

Different numbers of participants completed the symptoms questionnaire each week. Therefore, it would be misleading simply to report the number of incidences of ILI week by week. Instead, we consider an appropriate denominator, to allow us to estimate ILI incidence rates. The key to calculating this denominator is to determine who in the sample population is at risk of acquiring ILI. The most straightforward approach is to take as the denominator for any given week all those who completed the symptoms questionnaire that week. However, although we continually encouraged those without symptoms to report their lack of symptoms, it is highly probable that individuals were more motivated to complete the surveys on those weeks when they experienced symptoms; thus, one could argue that the denominator should include all flusurvey participants, whether or not they completed the symptoms questionnaire that week.

Another issue arises when we consider in more detail what is actually being measured; although we classify symptoms as "ILI" or "non-ILI", it is likely that during summer/autumn 2009 much of what was classified as ILI was in fact H1N1v influenza. If this is so, then participants will be unable to experience a second case of "ILI", and should be removed from the population considered to be at risk. We examine two possibilities: the first, in which we assume that participants could experience more than one case of ILI, and that they are immediately at risk of subsequent infection (i.e. a susceptible-infected-susceptible (SIS) infection); the second in which we assume that ILI represents H1N1v, and that participants are removed from the population at risk once they have experienced ILI (i.e. a susceptible-infected-removed (SIR) infection).

In summary, we have 4 different methods of calculating a plausible denominator: either SIS or SIR, and either assuming the weekly population or the total population to be at risk. In what follows, we describe these 4 possibilities as

• SIS week

• SIS sample

• SIR week

• SIR sample

Unless otherwise stated we use the SIS week assumption for incidence.

### Comparator estimates of incidence

**Figure 3:**
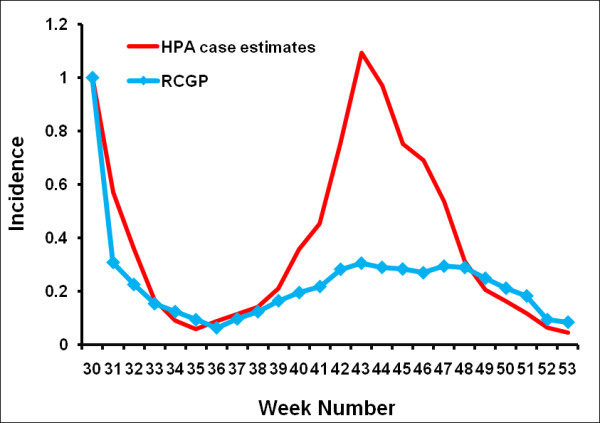
**Comparison of weekly ILI incidence as calculated by the RCGP with the weekly HPA case estimates**.

The standard measure of ILI incidence in England and Wales is the Royal College of General Practitioners Weekly Returns Service (RCGP). This is a sentinel GP-based scheme. A small fraction of the cases are swabbed and tested virologically to determine their infection status. During the H1N1 epidemic, the HPA also estimated weekly case numbers. These estimates took into account the fraction of cases attending GP surgeries and the National Pandemic Flu Service (NPFS - an internet and telephone based service that was set-up in July 2009 to help ease the burden on GPs and the NHS Direct) that were virologically confirmed. There was a marked difference in confirmation rates between the first and second wave of the 2009 epidemic, so that although the summer wave appeared to be much higher than the autumn wave according to the RCGP scheme, the estimated true height of the second wave was about equal to the first wave (Figure 3). The relative peak in deaths over the two waves closely matches the peaks in the HPA case estimates [29]. For these reasons we assume that the HPA case estimates give a more accurate picture of the true influenza incidence than the RCGP consultation rate (though it should be noted that the HPA case estimates are estimates of H1N1v influenza, and ignore other causes of ILI).

## Results

**Figure 4:**
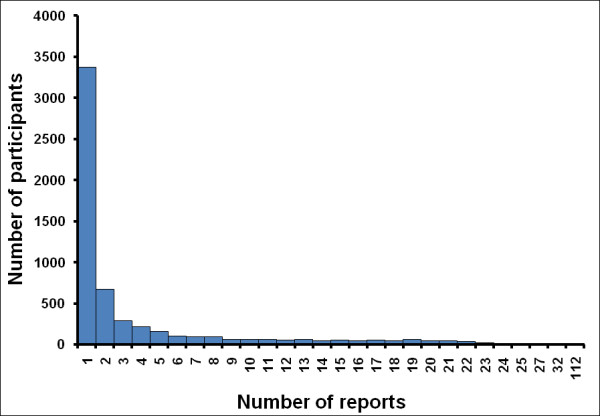
Total number of reports per participant in the crude (complete) dataset.

**Figure 5:**
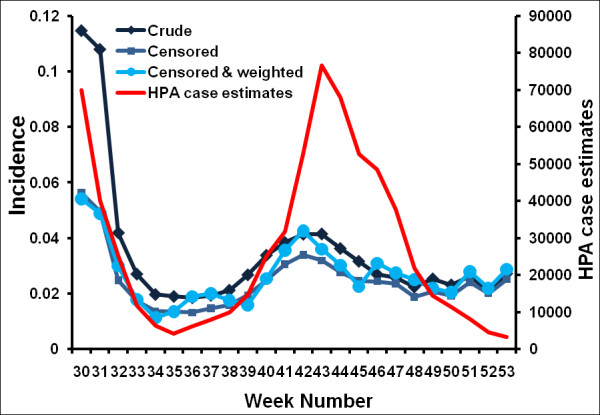
**Time series of the proportion of participants reporting ILI each week (using the GIS definition of ILI). **Three different flusurvey incidence curves are plotted: one that uses the crude (complete) dataset, one using the censored dataset (ignoring all participants' who participated only once) and one using the censored dataset and reweighting the population to account for demographic unrepresentativeness, compared with the cases estimated by the HPA [21]. All curves are plotted using a 3-week moving average; plots without this moving average can be found in the Supplementary material.

**Figure 6:**
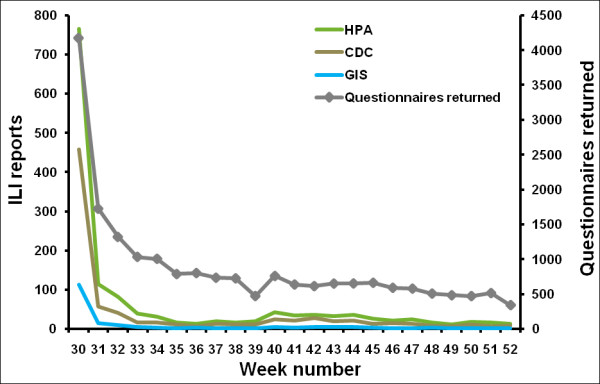
Comparison of the weekly symptoms questionnaires that were returned with the number of ILI reports for the same week, in the crude (complete) dataset.

We analysed the Flusurvey data from week 29 2009 up to week 53 2009. This dataset contained 5,738 participants out of whom 3,370 reported only once (Figure 4). Figure 5 (also see Additional file 2) demonstrates that this crude dataset considerably overestimates the relative height of the first peak when compared with the HPA case estimates, while the censored (and weighted) dataset of 2,369 people gives a better indication of the second peak. Figure 6 indicates that this may be due to many people being motivated to take part at the beginning of the survey because they themselves had flu-like symptoms. A Pearson's correlation test with the censored and weighted dataset against the HPA case estimates for week 30 to week 53 gives a value of 0.713 (P-value: 0.0001, 95% CI: 0.435, 0.867) as compared to the correlation coefficient of the censored (without weighting) dataset of 0.699 (P-value: 0.0001, 95% CI: 0.412, 0.860). The relative height of the two peaks when using the censored and weighted dataset is in reasonably close agreement with the relative heights of the two HPA case estimate peaks. In contrast the RCGP system reported a much higher first wave (Figure 3).

About 17% (391 out of 2,369) of our participants had ILI at least once, out of whom 29% (113 out of 391) said they were in the risk group, while 8% (33 out of 391) said they were health care workers. About 43% (170 out of 391) of our ILI cases claimed to have met an infectious contact in the week prior to their illness.

**Figure 7:**
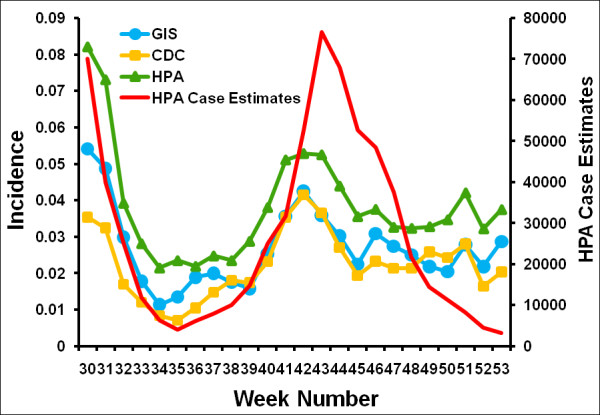
**Comparison of ILI incidence according to different definitions of ILI with the HPA's estimated ILI cases **[17,21,27,28]. As in Fig 3, the denominator used is those participants who completed the symptoms questionnaire on the week in question. All curves are plotted using a 3-week moving average; plots without this moving average can be found in the Supplementary material.

**Figure 8:**
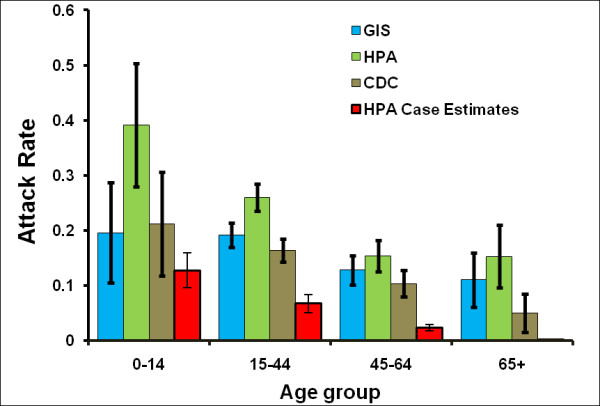
**The estimated attack rates using 3 different definitions of ILI, broken down into age groups, along with estimated attack rates by the HPA **[23]. The attack rates for the HPA estimated cases have been calculated based on Baguelin *et al*. [23] method.

On exploring the effect of using different definitions of ILI (Figure 7, also see Additional file 3), we see that all definitions follow the same broad pattern, with peaks in incidence in the summer and the autumn. As might be expected from the definitions, the incidence calculated using the HPA definition is higher than that calculated using the more stringent GIS and CDC definitions. A comparison of the overall attack rates as estimated from the Flusurvey data for England according to these various definitions with English attack rates calculated by Baguelin *et al*. [23], for the same time period, shows that the Flusurvey attack rates are significantly higher (Figure 8). Our overall attack rate according to the GIS definition [17] is 16% while the overall attack rate for England (HPA estimates) is 6% [23]. 1.9% of our sample reported visiting a GP for ILI over the time period (4% visited or telephoned the surgery) compared with a cumulative GP consultation rate for ILI of 1% in the RCGP system [21].

**Figure 9:**
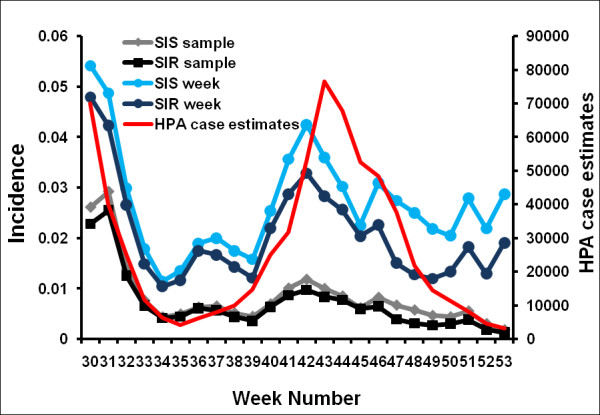
**Incidence according to different denominators compared with the case estimates of the HPA **[21]. All curves are plotted using a 3-week moving average; plots without this moving average can be found in the Supplementary material.

In Figure 9 (also see Additional file 4) we explore the impact of different ways of calculating the denominator in our incidence estimates. As we would anticipate, using the entire sample as the denominator results in a lower incidence estimate and a relatively higher second wave than when only using those participants who report in a given week. The fact that the SIS method produces a larger estimate of incidence than the SIR method suggests that some participants experienced repeated infections of ILI. The reinfection rates according to GIS (82/391) and HPA (111/520) definitions of ILI is 21% and for CDC definition is 17% (54/313).

## Discussion

Data from the UK suggests that the standard (GP-based) surveillance of ILI during the 2009 pandemic gave a distorted view of epidemic progress. In particular, the height of the summer peak was exaggerated in comparison to the autumn peak (Figure 3). This occurred because of changes in the pattern of access to care that occurred during the epidemic. For instance, data from the Flusurvey suggests that about 45% of those with ILI sought medical attention in July, dropping to around 20% in August, and recovering again to around 30% during the autumn peak (Brooks-Pollock E, Tilston N, Edmunds WJ, Eames KTD: **Re-estimating H1N1 cases numbers in England in light of changing health-seeking behaviour ***Submitted*). To get a clearer picture of the time course of the epidemic the HPA instigated random testing of patients accessing different health-care settings, which allowed the true numbers of cases to be estimated. However, this is expensive and induced further delays in the data stream. As is shown here, internet-based monitoring of ILI in the community can provide a direct and timely alternative - the relative size and timing of the peaks being close to the adjusted HPA case estimates. Because data were collected from people who did not attend health care, it is likely that internet-based surveillance captures a wider range of cases than traditional (i.e. GP-based) surveillance systems, and allows changes in utilisation of care to be monitored and accounted for in real-time. Internet-based surveillance is relatively quick and inexpensive to initiate, and is straightforward to scale up. However, as is also evident from this study considerable care must be taken to appropriately account for the different biases in the system.

The level of incidence observed is dependent on the definition of ILI and the denominator used. While there is a marked difference in the attack rates generated by different ILI definitions, all are higher than that estimated by a mathematical model [23]. There are a number of possible explanations for this observation: first, not everything that is diagnosed as ILI will have been H1N1v influenza - the fact that some individuals reported more than one episode of ILI illustrates this. Second, the survey may result in response biases; we have, through weighting the sample, attempted to adjust for demographic (age and gender) biases in response, but it is not possible to adjust for unknown confounders. In addition it is likely that individuals preferentially took part when they were infected. Although censoring the sample by removing those individuals from the analysis who only report on one occasion reduces the bias somewhat, as illustrated by the estimated incidence curves, it is likely that some remains. In presenting the use of different denominators for incidence estimates we have explored two possibilities: one that assumes that completion of the survey in a given week is independent of symptom status, and the other that assumes that all participants with ILI in a given week will complete the survey that week. It is likely that reality lies somewhere in between, and further work is clearly needed to determine the nature of this response bias and how to adjust for it.

In making comparisons between Flusurvey estimates and HPA case numbers, it must be noted that the HPA figures are themselves only estimates, based on recorded treatment seeking behaviour (contact with GPs and use of the National Pandemic Flu Service), adjusted for antiviral positivity and a constant which represents the fraction of cases that consult GPs. Given that treatment-seeking behaviour changed over the course of the pandemic, there is no authoritative time series against which to compare Flusurvey incidence estimates. Furthermore, because our internet-based survey measures ILI, we would expect the difference between the Flusurvey and HPA case estimates to be largest when pandemic H1N1v was rare. This, indeed, was the case, with the Flusurvey (and RCGP) recording continuing ILI activity through December, whereas the HPA case estimates (which account for the fraction that were confirmed as H1N1v) were very low at this point (Figure 5).

It is likely that systems such as the UK Flusurvey will be increasingly used in the future, for both pandemic and seasonal influenza, as they are a relatively low-cost method of collecting ILI data, and allow access to cases that do not necessarily seek formal care. However, these systems are novel and need to be properly evaluated. One suggestion for validation would be to virologically test patients with ILI symptoms (perhaps by sending out self-swabbing packs to participants who report ILI symptoms), similar to the testing scheme implemented for the NPFS during the 2009 epidemic.

## Conclusions

Internet-based surveillance has the potential to capture a wider range of cases than traditional (GP-based) surveillance systems, as well as to track changes in health-care attendance patterns in real-time. Our results suggest that trends in incidence can be captured by such systems perhaps even more reliably than standard GP-based systems, but it remains unclear how accurate they are for estimating the absolute level of incidence. Internet-based surveillance does not offer a replacement of traditional GP-based methods, but can provide an important adjunct, allowing the collection of valuable additional information.

## Competing interests

The authors declare that they have no competing interests.

## Authors' contributions

NLT was involved in editing of the UK Flusurvey website, analysis of data, presentation of results and preparation of the manuscript. DP and TE were involved in the technical aspects of the project. KTDE and WJE conceived of the study, participated in its design and coordination, participated in the management, design and editing of the website and preparation of the manuscript. All authors read and approved the final manuscript.

## Pre-publication history

The pre-publication history for this paper can be accessed here:

http://www.biomedcentral.com/1471-2458/10/650/prepub

## Supplementary Material

Additional file 1 **Map from the flusurvey showing the geographical spread of participants**. This shows the approximate location of flusurvey users: red points indicate people with influenza-like symptoms, blue indicates people with other respiratory symptoms and green indicates people who do not have respiratory symptoms. Click here for file

Additional file 2 **Time series of the proportion of participants reporting ILI each week (using the GIS definition of ILI), without moving averages**. Three different flusurvey incidence curves are plotted: one that uses the crude (complete) dataset, one using the censored dataset (ignoring all participants' who participated only once) and one using the censored dataset and reweighting the population to account for demographic unrepresentativeness, compared with the cases estimated by the HPA [21]. Click here for file

Additional file 3 **Comparison of ILI incidence according to different definitions of ILI with the HPA's estimated ILI cases **[17,21,27,**28**], **without moving averages**. As in additional file 1, the denominator used is those participants who completed the symptoms questionnaire on the week in question. Click here for file

Additional file 4 Incidence according to different denominators compared with the case estimates of the HPA, without moving averages. Click here for file
