# Supplementary figures and images for: Internet-based surveillance of Influenza-like-illness in the UK during the 2009 H1N1 influenza pandemic
Source: BMC Public Health. 2010 Oct 27;10:650. doi: 10.1186/1471-2458-10-650 (PMC2988734; doi:10.1186/1471-2458-10-650)

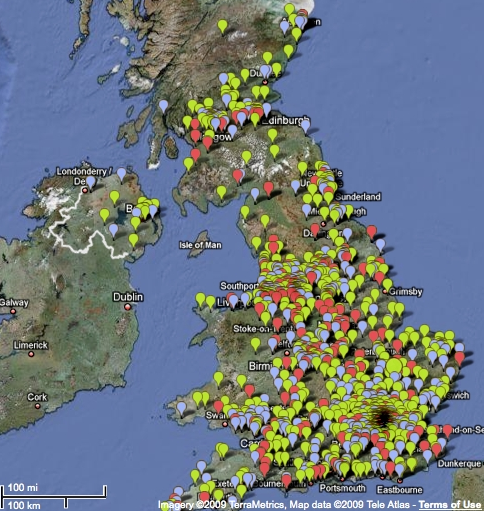

Supplement: Additional file 1 — Map from the flusurvey showing the geographical spread of participants. This shows the approximate location of flusurvey users: red points indicate people with influenza-like symptoms, blue indicates people with other respiratory symptoms and green indicates people who do not have respiratory symptoms. [file 1471-2458-10-650-S1.BMP]

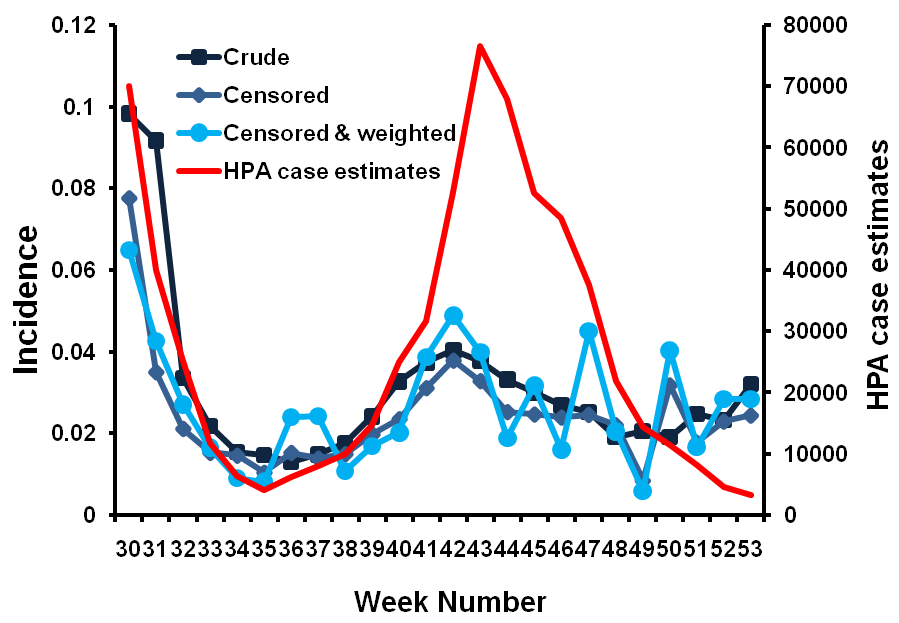

Supplement: Additional file 2 — Time series of the proportion of participants reporting ILI each week (using the GIS definition of ILI), without moving averages. Three different flusurvey incidence curves are plotted: one that uses the crude (complete) dataset, one using the censored dataset (ignoring all participants' who participated only once) and one using the censored dataset and reweighting the population to account for demographic unrepresentativeness, compared with the cases estimated by the HPA [21]. [file 1471-2458-10-650-S2.PNG]

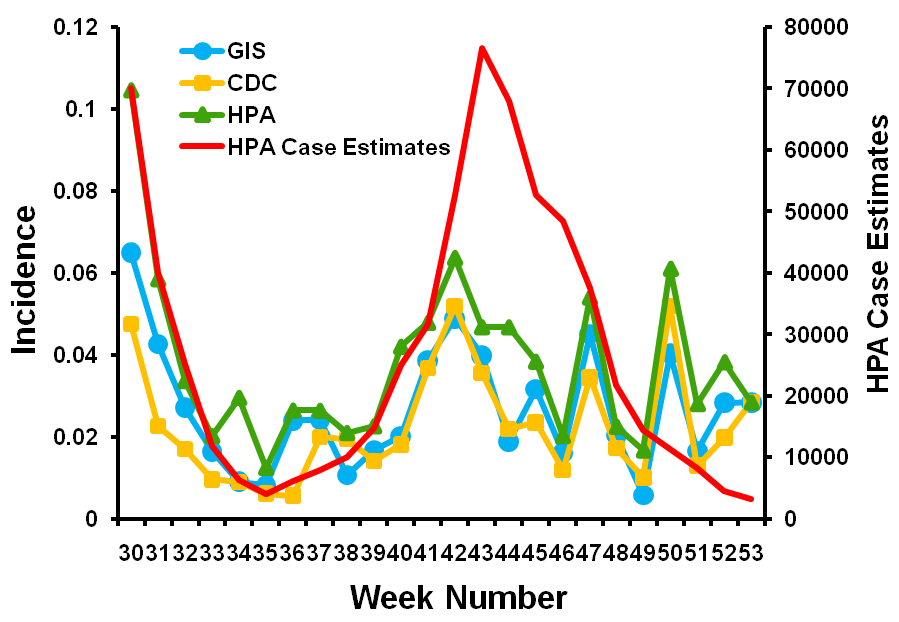

Supplement: Additional file 3 — Comparison of ILI incidence according to different definitions of ILI with the HPA's estimated ILI cases [17,21,27,28], without moving averages. As in additional file 1, the denominator used is those participants who completed the symptoms questionnaire on the week in question. [file 1471-2458-10-650-S3.PNG]

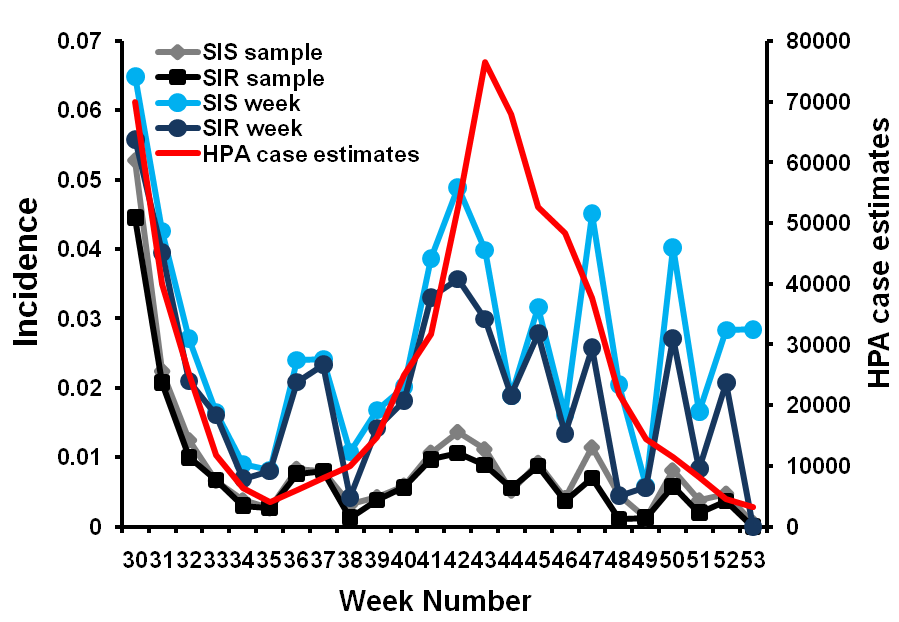

Supplement: Additional file 4 — Incidence according to different denominators compared with the case estimates of the HPA, without moving averages. [file 1471-2458-10-650-S4.PNG]
